# Menstrual cycle length trajectories and their health correlates across the reproductive lifespan

**DOI:** 10.21203/rs.3.rs-10629991/v1

**Published:** 2026-09-02

**Authors:** Makiko Mitsunami, Elizabeth Peebles, Boya Zhang, Brent A. Coull, Tamarra James-Todd, Jorge E. Chavarro, Jaime E. Hart, Francine Laden, Shruthi Mahalingaiah

**Affiliations:** National Center for Child Health; Harvard T.H. Chan School of Public Health; Harvard T.H. Chan School of Public Health; Harvard T.H. Chan School of Public Health; Harvard T.H. Chan School of Public Health; Harvard T.H. Chan School of Public Health; Harvard T.H. Chan School of Public Health; Harvard T.H. Chan School of Public Health; Harvard T.H. Chan School of Public Health

**Keywords:** menstrual cycle length, menstrual irregularity, reproductive health, polyendocrine metabolic ovarian syndrome (PMOS) metabolic syndrome, longitudinal studies

## Abstract

Although the menstrual cycle is a vital sign for women’s health, research investigating menstrual cycle length (MCL) trajectories across reproductive life is scarce, often categorizing just two patterns: regular and irregular.

We used latent class mixed models (LCMMs) to identify MCL trajectories among 9,332 females enrolled in the Growing Up Today Study, a United States prospective cohort followed from 1996–2023. Participants who self-reported their MCL three or more times were included in the analysis. Additional demographic and health information collected from surveys were included in the descriptive statistics, stratified by trajectory classification.

The best fitting LCMM identified four MCL trajectories: “always regular” (94.2% of study population), “early long transition” (2.5%), “late long transition” (3.0%), and “long to short transition” (0.2%). Those classified in the “early long transition” trajectory had a higher proportion of adulthood obesity and polyendocrine metabolic ovarian syndrome (PMOS); “late long transition” individuals had higher proportions of childhood and adulthood obesity, hirsutism, severe acne in adolescence, PMOS, hypothyroidism, hypertension, and endometriosis.

The identified MCL trajectories serve as a proof of concept for identifying menstrual irregularity as a signal of disease markers warranting clinical evaluation, though further validation is needed before clinical application.

## Introduction

Menstrual cycle length (MCL) is the number of days between the start of one period to the start of the next. It varies from menarche to menopause within a range. Its regularity can be a proxy for a multitude of health conditions, such as ovulation disorders; gynecological ailments; and chronic diseases, including cardiovascular disease, diabetes, cancer, cognitive function, and premature mortality.^1–8^ Foundational studies on menstrual cycle variability conducted in the 1960s established that most women reported a MCL ranging from 15–45 days. The current definitions of regular MCLs vary; the American College of Obstetricians and Gynecologists (ACOG) defines a regular MCL as 21–35 days,^9^ whereas the International Federation of Gynecology and Obstetrics (FIGO) defines a regular MCL as 24–38 days.^10^ Ovulatory disorders, such as polyendocrine metabolic ovarian syndrome (PMOS), primary ovarian insufficiency (POI),^11^ functional hypothalamic amenorrhea (FHA),^12^ and hyperprolactinemia as well as other endocrine disorders, such as obesity and thyroid disease, can alter MCL by affecting the function of the hypothalamic-pituitary-ovarian (HPO) axis.^13^ These conditions have varied effects on MCL and regularity. Other factors influencing MCL include age at menarche, parity, adiposity, genetics, age, health conditions, medications, and environmental factors and stressors.^14^ Previous literature also suggests MCL varies by race and ethnicity.^15,16^

MCL changes across the reproductive lifespan. It is well established that cycle length variability increases immediately following menarche and shortly before menopause.^17^ Existing literature often cross-sectionally assessed the average or modal MCL across the lifespan.^18–23^ Such approaches capture cycle-to-cycle variability within individuals but fail to capture longer-term, individual MCL trajectories across the reproductive lifespan. Only two studies have attempted to classify intraindividual MCL patterns throughout the reproductive lifespan, both using data from the Tremin Study Research Program on Women’s Health, a foundational prospective women’s health study started in the 1930s that may not reflect modern day conditions.^24,25^

Our prospective study aimed to elucidate MCL trajectories across the reproductive life course using latent class mixed models (LCMM) among a United States (US) population. By identifying MCL trajectories, we can move beyond the regular-irregular binary to better understand of menstrual cycle complexity and eventually understand the linkage between trajectories and health conditions, maximizing the menstrual cycle’s utility as a vital sign across the life course.

## Results

### Sample Characteristics

The Growing Up Today Study (GUTS) is a prospective US cohort comprising children of participants in the Nurses’ Health Study II, enrolled at ages 9–16 years across two cohorts (GUTS1 in 1996; GUTS2 in 2004) and followed longitudinally through 2023 via periodic questionnaires. Among 9,332 GUTS participants who reported at least three MCLs from 2003 to 2023, we included a total of 48,494 MCL reports, with a median (IQR) of 5 (4, 6) per participant. The mean (SD) age at baseline (1996 or 2004) was 12.2 (1.9) years, and 8,769 participants (94.8%) were non-Hispanic White (Table 1). The MCL survey questions asked about the participants’ average MCL when not pregnant, breastfeeding, or using birth control pills. By 2023, approximately 90% of eligible participants had ever used oral contraceptives. Compared to participants excluded from the primary analysis due to lack of MCL data or hysterectomy (Figure S1), those included were more likely to be born earlier (Table S1). Age at baseline and frequency of reporting irregular MCLs did not differ between the included and excluded participants, and the proportion of irregular cycles was similar (14.4% vs. 14.6%; Table S1).

#### Latent Class Mixed Model

Latent class mixed models (LCMMs) were used to identify distinct MCL trajectory patterns across the reproductive lifespan, accommodating the ordinal nature of the MCL outcome and intermittent missing data. As this was an exploratory analysis, the maximum number of latent classes was not pre-specified. Of the five models fitted (two through five latent classes), the five-class model failed to converge. The LCMM with four latent classes had the lowest Bayesian Information Criterion (BIC) (120,501.47) with sufficient average posterior probabilities (0.67–0.93) compared to the two (120,533.86) and three (120,561.28) latent class models (Fig. 1, Figure S2).

Figure 1 illustrates the distribution of MCL reports across the reproductive lifespan overlayed with the fitted curves of the four identified trajectories using the four latent class LCMM. Trajectory 1 encompassed participants with “always regular” MCLs (94.2%) and exhibited minor expected fluctuations across the duration of follow up: a slightly longer cycle in the teenage years and early thirties, which shortens as participants approach their forties. Three irregular trajectories (Trajectory 2, 3 and 4) were identified: Trajectory 2 (“early long transition”, 2.5%) described regular, short cycles that transition to long, irregular menstrual cycles or no period in participants’ mid-twenties, Trajectory 3 (“late long transition”, 3.0%) was similar to Trajectory 2, but the shift occurred in the participants’ early thirties, and Trajectory 4 (“long to short transition”, 0.2%) described long cycles that transitioned to short cycles during the participants’ twenties and thirties. Within each trajectory, the proportion of self-reported study defined normal length MCLs (21–39 days) was 87.6% for Trajectory 1, 42.9% for Trajectory 2, 67.3% for Trajectory 3, and 51.4% for Trajectory 4 (Table 1). When two- or three- latent class models were applied, only two trajectories were identified (Trajectory 1 and Trajectory 2).

Those who reported their menstrual cycle length (MCL) on three or more questionnaires from 2003–2023 were included (number of subjects: 9,332, number of reports: 48,494). The plot illustrates the MCL distribution, colored to the assigned trajectory pattern, overlayed with fitted curves based on the latent class of MCL (ordinal outcomes). The fitted curves represent the estimated average MCL for each class at a given age. The different trajectories are categorized into Trajectory 1: “always regular”, Trajectory 2: “early long transition”, Trajectory 3: “late long transition”, and Trajectory 4: “long to short transition.” The x-axis represents age (years) at menstrual cycle reports. The Bayesian Information Criterion (BIC) is displayed.

#### Participant Characteristics by Trajectory

Tables 1 and 2 present demographic and reproductive characteristics across four trajectories identified by the best fitting LCMM. Trajectory 4 had notably fewer participants (n = 19), limiting comparisons. Participants in Trajectory 3 were born earlier than those in Trajectory 1. Adulthood obesity was more prevalent in Trajectory 2 (32.1%) and Trajectory 3 (36.5%) compared to Trajectory 1 (25.6%). However, childhood obesity was most common in Trajectory 3 (13.5%) and Trajectory 4 (10.5%). Trajectory 3 also showed the earliest age at menarche (12.6 ± 1.1 years), compared to Trajectories 1, 2, and 4 (all 12.8 ± 1.1–1.2 years; p = 0.04; Table 1).

In 2019, when pregnancy history was assessed, nulliparity was more frequent in Trajectory 2, and the average age at first childbirth was about one year younger in both Trajectory 2 (27.5 ± 4.0 years) and Trajectory 4 (27.5 ± 5.1 years) compared to Trajectory 1 (28.9 ± 3.6 years) and Trajectory 3 (28.5 ± 3.5 years; Table 1).

Possible FHA was defined by the presence of all of the following at any point during follow-up: oligo-amenorrhea, underweight body mass index (BMI) in childhood or adulthood, or excessive exercise, and the absence of PMOS-associated traits or thyroid disease. Compared to Trajectory 1, both Trajectory 2 and Trajectory 4 exhibited a higher prevalence of possible FHA (Trajectory 2: prevalence ratio [PR] = 3.94; 95% CI: 2.48–6.27, Trajectory 4: PR = 5.47; 95% CI: 1.92–15.55). Self-reported PMOS was more prevalent in Trajectories 2 (PR = 1.37; 95% CI: 1.06–1.76) and 3 (PR = 2.06; 95% CI: 1.35–3.14). PMOS-associated traits, defined as self-reported PMOS, severe adolescent acne, or hirsutism, were more prevalent in Trajectories 3 (41% higher risk; 95% CI: 12–78%) and 4 (76% higher risk; 95% CI: 20–159%) relative to Trajectory 1. Furthermore, Trajectory 3 had a higher prevalence of endometriosis (13.5%) compared to the other groups (Table 2).

#### Sensitivity Analysis

The first sensitivity analysis excluded MCLs reported during pregnancy, defined as the period from estimated conception through six months post-delivery or three months after any pregnancy loss, resulting in data from 5,994 participants with 29,301 MCL observations (Figure S3). While Trajectory 4 was not observed, the LCMMs with two to five classes yielded trajectories comparable to the main model. Trajectory 1 and Trajectory 3 were consistently identified, along with a more differentiated subgroup of Trajectory 2 as the number of latent classes increased.

The second sensitivity analysis excluded MCLs reported in questionnaire cycles where concurrent hormonal contraceptive use was indicated, yielding data from 3,852 participants with 15,614 MCL observations (Figure S4). The LCMM with two latent classes showed the lowest BIC. The LCMMs with two and three classes demonstrated similar trajectories as Trajectory 1 and Trajectory 3 observed in the main model. The LCMM with four latent classes identified three trajectories: one was consistent with Trajectory 1, and two were not observed in the main analysis but may be subgroups of regular trajectories. The LCMM with five latent classes identified five trajectories that included Trajectory 1 and Trajectory 3, two subgroups of regular trajectories, and an always-short trajectory.

## Discussion

Our study is the first, to our knowledge, to utilize LCMMs to identify MCL trajectories throughout the reproductive lifespan within the same individuals. We identified four distinct MCL trajectories: Trajectory 1 (“always regular”), Trajectory 2 (“early long transition”), Trajectory 3 (“late long transition”), and Trajectory 4 (“long to short transition”). Most participants (94.2%) were categorized within the “always regular” pattern (Trajectory 1) and the remaining 5.8% were categorized among the irregular pattern trajectories (Trajectories 2–4). Our findings suggest that these trajectories represent population-level patterns of MCL change rather than proxies for specific diagnoses, and that irregular trajectories should be interpreted as signals warranting clinical evaluation rather than as diagnostic criteria.

Research on MCL variability began with large menstrual diary cohorts in the 1960s and 1970s.^17,18,21,26^ Research continued with short-term menstrual diary studies by the World Health Organization,^27,28^ other population-based studies,^29,30^ and now includes research utilizing large-scale data from menstrual cycle tracking smartphone applications.^16,31–34^ Many of these studies utilized cross-sectional approaches to describe gross changes in menstrual patterns throughout reproductive life. While there are a couple of studies that have prospectively monitored participants over the long term (up to 39 years), more than half of the participants were followed for only a few years, and the number of participants with longitudinal data was relatively modest (~ 700 participants).^18,21–24,35^ Similar to our findings, previous literature indicates that for those with regular cycles, MCL variability is most pronounced immediately following menarche and shortly before menopause,^17,35^ and that the average MCL increases in individuals’ early 20s and decreases by 1–2 days in individuals’ early 40s, with reduced MCL variability.^17,33^

Minimal research has attempted to classify MCL beyond the binary regular-irregular categorization. One study using the Tremin Trust data proposed five classifications of menstrual histories based on MCL and variability.^24^ The distribution of these five categories suggests that oscillating and erratic shifts in MCL, somewhat similar to Trajectory 4, are the most common. However, Trajectory 4 was the least populated trajectory in our main model. Another study proposed six categories of the menopausal transition using eight parameters, including the mean and variance of change points and menstrual length at age 35 and the changes in MCL mean and variance before and after these points.^25,36^ However, our study describes the variation of different MCL trajectories at the population level using LCMMs, which capture both individual-level temporal changes and population-level heterogeneity. This methodological approach addresses a major limitation in existing literature, where studies with only short-term follow-up may miss critical shifts in MCL trajectories. For example, distinguishing between Trajectory 2 and Trajectory 3 depends on when the transition from normal to longer cycle length occurs. Additionally, cross-sectional or short-term assessments risk the potential of misclassification. Among GUTS participants categorized as “always regular,” approximately 12% ever reported irregular MCLs. A cross-sectional study may not capture enough data to understand a participant’s appropriate MCL classification. By leveraging LCMMs and longitudinal data, our study overcomes these challenges, providing a more comprehensive and nuanced understanding of MCL trajectories.

MCL trajectories have the potential to support early identification of individuals warranting clinical evaluation for reproductive, endocrine, and cardiometabolic conditions. Trajectories are already integrated throughout clinical practice to characterize disease progression and enhance risk prediction by monitoring patient changes over time.^37^ For instance, pediatricians track BMI-for-age on growth charts during annual visits, and obstetricians monitor gestational weight gain throughout pregnancy to assess risk for conditions such as obesity or gestational diabetes. The menstrual cycle represents an underutilized health metric that, like vital signs, could serve as an important tool for predicting clinical outcomes. The present study represents a preliminary step toward formalizing MCL trajectory monitoring as a framework for identifying individuals who may benefit from further clinical evaluation, while acknowledging that trajectory patterns alone are insufficient for clinical diagnosis.

It is important to acknowledge that our results suggest that the observed MCL trajectories likely reflect heterogeneous underlying biological processes rather than distinct, mutually exclusive disease phenotypes. Although irregular trajectories may be associated with ovulatory dysfunction such as FHA, POI, and PMOS, no single trajectory corresponds exclusively to one condition, as multiple pathological and physiological pathways can produce similar MCL patterns. Therefore, an irregular MCL trajectory should be interpreted as a signal warranting comprehensive clinical evaluation rather than as a diagnostic criterion.

Building on this interpretive framework, each irregular trajectory exhibited a distinct pattern of associated health characteristics that highlights specific clinical domains warranting attention, rather than mapping onto a single diagnosis. Compared to Trajectory 1, the other trajectories demonstrating irregular cycle patterns may represent a clinically heterogenous group.

Trajectory 2 (“early long transition”) demonstrated contributions from multiple clinical features and conditions. Self-reported physician-diagnosed PMOS was more prevalent than in Trajectory 1, while PMOS-associated traits (self-reported PMOS, severe adolescent acne, or hirsutism) were not. Additionally, possible FHA and hypothyroidism were both elevated. Individuals in Trajectory 2 may represent a clinically heterogeneous group with similar abnormality in cycle length pattern warranting evaluation of hypothalamic function, thyroid status, and androgenic features.

Participants in Trajectory 3 (“late long transition”) demonstrated a higher prevalence of endometriosis and cardiometabolic risk factors, including obesity from childhood, PMOS-associated traits, and hypercholesterolemia, compared to Trajectory 1. The presence of childhood obesity and androgenic features in adolescence raises the possibility that metabolic predisposition may precede the onset of menstrual irregularity in this group, consistent with prior evidence of 1) the association between irregular cycles and cardiometabolic disease, and 2) shared inflammatory pathways between endometriosis and cardiometabolic conditions.^38–41^

Trajectory 4, though limited by small sample size (n = 19), showed a premature pattern of cycle shortening alongside elevated PMOS-associated traits, despite low self-reported PMOS. Premature cycle shortening of this nature may suggest a trajectory toward POI, which affects fewer than 5% of the population,^11^ although distinguishing this from other causes would require objective hormonal and ovarian reserve assessment. Given the small sample size, all findings for this group remain hypothesis-generating and warrant investigation in larger cohorts.

This study has several limitations. First, we collected self-reported usual MCL as an ordinal variable, with only one response per GUTS questionnaire cycle (ranging from one to seven years apart). This response method and cadence could lead to measurement errors and misclassification. However, since participants were asked to retrospectively report their average MCL over an extended period rather than recording exact cycle lengths for each individual cycle, collecting this variable as a continuous measure would have introduced false precision, as any numerical response would itself have been a subjective approximation. Therefore, a continuous variable analysis was not feasible given the nature of the data collected. Additionally, since the “no period” questionnaire answer option was introduced in 2011, participants experiencing amenorrhea might have selected “too irregular to estimate.” Nonetheless, categorizing menstrual cycle lengths has been shown to be accurate and could help capture the natural within individual variability.^17,35^ An additional limitation is that the characterization of possible FHA and PMOS-associated traits relied solely on self-reported biological characteristics rather than clinical measurements such as blood tests or imaging. While self-reported symptoms are recognized components of diagnostic criteria, the absence of hormonal profiling, ovarian reserve measurements (e.g., anti-Müllerian hormone levels or antral follicle count), and imaging data limits the biological interpretability of the identified trajectories. Additionally, the relatively small number of participants in Trajectory 4 (n = 19) limits the statistical power and reliability of findings specific to this group, and these results should be interpreted with caution. Although the five-class model was explored, it did not converge, further supporting our decision to retain the four-trajectory model as the most stable and interpretable model given the available sample size.

Another limitation is the high proportion of hormonal contraceptive usage: according to the National Survey of Family Growth (NSFG), 88% of sexually active women in the US reported contraceptive use between 2014 and 2016.^42^ We asked participants to report their MCL when not influenced by pregnancy, breastfeeding, or hormones such as birth control pills. For participants who have been using hormonal contraceptives for many years, their retrospective report of MCL may not reflect their actual cycle at the time of assessment. Furthermore, the 2006–2008 NSFG revealed that 28% of individuals use oral contraceptive pills to address irregular menstruation.^43^ Therefore, excluding all contraceptive users could introduce a selection bias by disproportionately retaining individuals with regular menstrual cycles. Additionally, our sensitivity analysis excluding concurrent hormonal contraceptive use supports our main model findings, although the reduced sample size (approximately 40% of the main analysis) limits comparability. Lastly, GUTS is a US cohort, so it reflects Western health and lifestyle standards which limits generalizability.^44^ Future research in other populations using this statistical method may demonstrate more refined trajectory patterns.

This study’s strengths include being based in a large, prospective cohort with 20 years of follow-up and linked maternal data. Especially in the US, contraceptive use is widespread, so collecting large-scale data without the influence of contraceptives will be increasingly complex in the future. This study is the first approach to capture MCL trajectories across the reproductive lifespan, and it serves as a preliminary attempt to bridge longitudinal MCL characteristics with reproductive and cardiometabolic health research. Our findings provide clinical implications by demonstrating that irregular MCL trajectory patterns are associated with a heterogeneous range of reproductive and cardiometabolic health characteristics, underscoring their potential utility as signals for clinical evaluation and follow-up.

In conclusion, we identified MCL trajectories across the reproductive lifespan, beyond the standard regular-irregular binary, which are possibly associated with cardiometabolic risk factors. These trajectory patterns can be used to better understand the health of the individual, especially for those with irregular cycles. These trajectories need to be validated in larger and more diverse populations. Recent advances in digital health technologies, which enable the collection of large-scale, cycle-level menstrual data from menarche to menopause through mobile applications and other digital tools, can be used to provide sufficient data to run future LCMMs.

## Methods

### Study design

We analyzed longitudinal data from 9,332 females enrolled in the Growing Up Today Study (GUTS), a US prospective cohort followed from 1996–2023 through periodic questionnaires. This study was conducted in accordance with all applicable laws and institutional guidelines, and received ethical approval from the Harvard Longwood Campus Institutional Review Board (#IRB23–0479) and the Mass General Brigham Institutional Review Board (1999P002104). Informed consent was obtained from all participants. For participants in the Growing Up Today Study (GUTS I & II), who were 9–15 years of age at enrollment, implied informed consent was obtained from their mothers, and assent was provided by participants themselves by returning completed questionnaires. All GUTS participants have since reached adulthood, and consent is implied through the return of questionnaires

#### Participants

GUTS is a prospective cohort of recruited children of female nurse participants in the Nurses’ Health Study II (NHSII),^45,46^ comprising two cohorts: GUTS1 (enrolled in 1996, ages 9–14 years at baseline) and GUTS2 (enrolled in 2004, ages 9–16 years at baseline). GUTS1 questionnaires were sent in 1996 (baseline), 1997, 1998, 2000, 2001, 2003, 2005, 2007, and 2010, while GUTS2 questionnaires were sent in 2004 (baseline), 2006, 2008, and 2011. The two cohorts merged in 2013, and questionnaires were sent to the combined cohort in 2013, 2014, 2015, 2016, 2019, 2021, and 2023. Of 27,805 GUTS participants, 15,044 reported female sex at birth; 9,332 participants (62%) were eligible for the analysis, having reported their MCL at least three or more times. MCL data after surgery were excluded for participants who self-reported a hysterectomy (2019 or 2021 questionnaires). A participant flow diagram is shown in Figure S1.

### Outcomes

MCL was assessed using survey questions asking about the current interval from the first day of your period to the first day of your next period with the categorical answer options of < 21 days, 21–25, 26–31, 32–39, 40–50, and 51 + days or too irregular to estimate (GUTS1: 2003, 2005, 2007; GUTS2: 2008, 2011; combined cohort: 2013, 2015, 2016, 2023). The option of “no period” was added in 2011. Question wording varied slightly over time (Table S3), but overall, the questions asked about participants’ average MCL when they were not pregnant, breastfeeding, or using birth control pills. We dichotomized MCL into normal length (a reported MCL between 21–39 days) and abnormal length (a reported MCL less than 21 days, greater than 40 days, too irregular to estimate, or no period).

### Covariates

Participants reported their birth date, age, race and ethnicity, and menstrual status (menarche). BMI was calculated using participants’ height (until 2011) and weight (all cycles). We defined childhood (< 20 years old) underweight as fifth percentile of the CDC age categorized BMI and childhood obesity using the International Obesity Task Force criteria;^47^ adulthood (≥ 20 years old) underweight and obesity was defined as a BMI < 18.5kg/m^2^ and ≥ 30kg/m^2^, respectively. Strenuous recreational activity (e.g., running, aerobics, lap swimming) was assessed using categorical variables in 2015. Excessive exercise was defined as ≥ 11 hours/week and low physical activity as < 2 hours/week.

Pregnancy history was collected in 2019. Contraceptive use (oral, intramuscular, subdermal, transdermal, vaginal) was reported starting in 1999 (GUTS1) and 2006 (GUTS2).

Participants self-reported physician-diagnosed PMOS (queried as polycystic ovary syndrome [PCOS] in all questionnaires, as data collection preceded the adoption of updated terminology), endometriosis, fibroids, hypertension, diabetes mellitus, hypercholesterolemia, hypothyroidism, and Graves’ disease/hyperthyroidism from 2010 to 2021. From 1999 to 2011, participants reported acne presence, acne severity, and whether they used oral contraceptives due to acne. Severe acne in adolescence was defined as self-reported severe acne or oral contraceptive use due to acne under the age of 19. Participants reported their chin, upper, and lower abdomen hair distribution in 2014 and 2016. The simplified Ferriman-Gallwey score of four or greater was considered hirsutism.^48^ Mothers of participants self-reported PMOS from 1989 to 2001 in the NHSII.

### Statistical analysis

We used LCMMs to identify MCL trajectories.^49^ Because the number of MCL trajectories was unknown a priori, we fitted LCMMs with two-five latent classes and compared model fit. We first fitted the LCMM of a single class, using MCL as the ordinal outcome. Age at MCL report, birth year, and their interaction were included as fixed effects. Because MCL was ordinal, we used a threshold link function, modeling the observed categories as arising from an underlying continuous process. Age at MCL report was calculated from questionnaire return month and self-reported birth month. The seven categories from the questionnaire response options (< 21, 21–25, 26–31, 32–39, 40–50 days, 51 + days or too irregular to estimate, and no period) were used as longitudinal outcomes. LCMM can accommodate intermittent missingness in outcome measurements across subjects.

Next, we fit models from two to five latent classes with the threshold link function and calculated the posterior probability of classification and the proportion of individuals classified in each trajectory. To compare the different models, we calculated the Bayesian Information Criterion (BIC).

We compared demographic and reproductive characteristics across the identified trajectories. Possible FHA was defined as meeting all of the criteria at any point during the study period: 1) ever reporting oligo-amenorrhea (MCL 40–50 days, 51 + days, cycles too irregular to estimate, or no menstrual period) between 2003 and 2023; 2) underweight in childhood or adulthood, strenuous physical activity > 11hr/week, and 3) the absence of PMOS traits (self-reported PMOS, severe adolescent acne, and hirsutism), and the absence of hypothyroidism or hyperthyroidism.^12^

### Sensitivity analysis

We conducted two sensitivity analyses. First, we ran LCMMs with two–five latent classes excluding those who reported concurrent hormonal contraceptives use. Second, we ran LCMMs with two–five latent classes excluding MCLs reported during a pregnancy, defined by the estimated time of conception to six months post-delivery or three months post-miscarriage, stillbirth, induced abortion, or ectopic pregnancy. For both analyses, those who contributed three or more MCLs after each exclusion were included. We used R v4.3.2 software and LCMM package v2.2.1 to identify trajectories and SAS version 9.4 (SAS Institute Inc., Cary, NC) for the remaining analysis.

## Supplementary Material

Supplementary Files

This is a list of supplementary files associated with this preprint. Click to download.

- ScientificReportsSupplementary08072026.docx

## Figures and Tables

**Figure 1:**
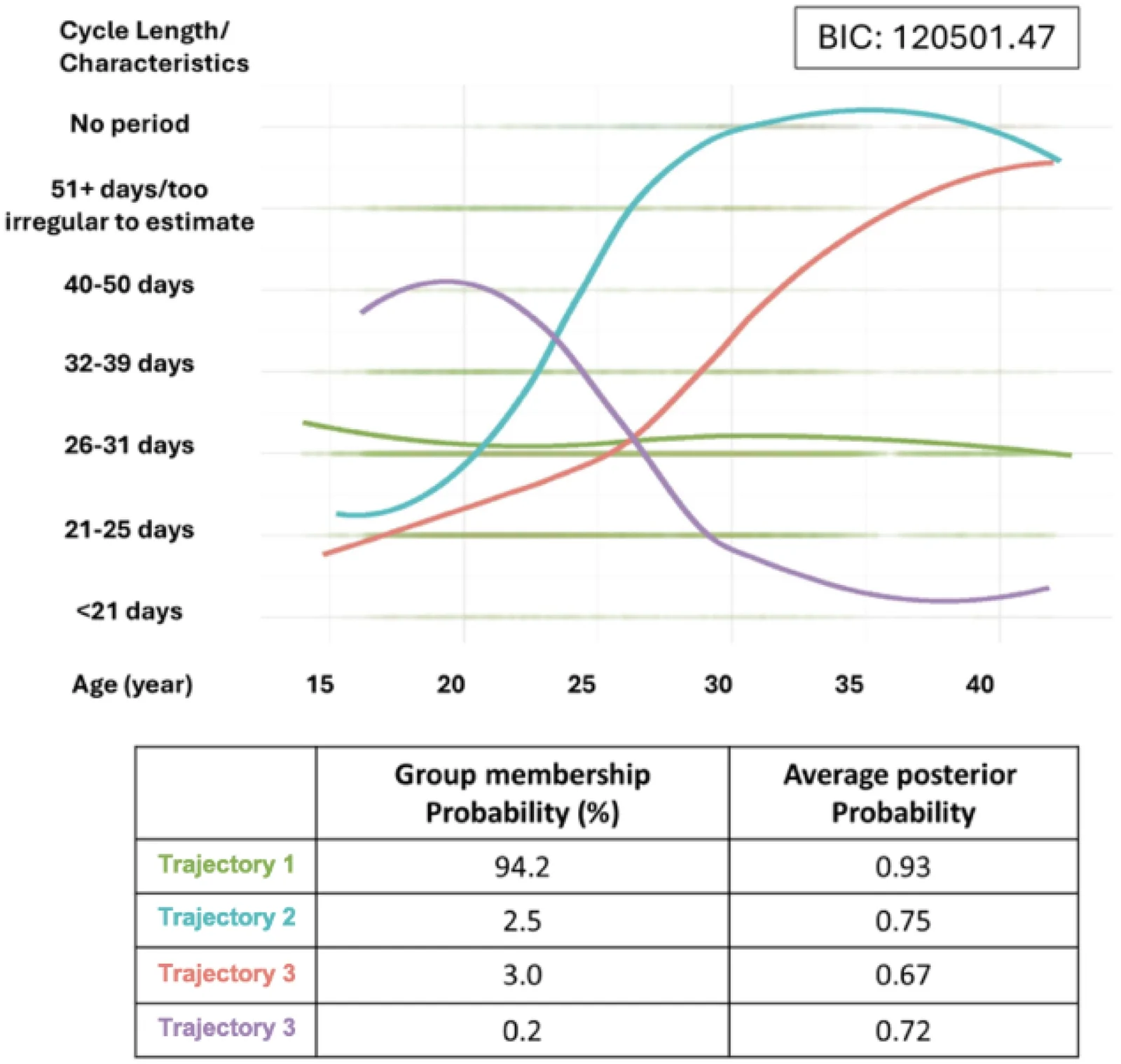
Latent class mixed model (LCMM) with four latent classes for self-reported categorical menstrual cycle lengths (MCLs) throughout the reproductive lifespan among participants in the Growing Up Today Study (GUTS) (1996–2023). Those who reported their menstrual cycle length (MCL) on three or more questionnaires from 2003–2023 were included (number of subjects: 9,332, number of reports: 48,494). The plot illustrates the MCL distribution, colored to the assigned trajectory pattern, overlayed with fitted curves based on the latent class of MCL (ordinal outcomes). The fitted curves represent the estimated average MCL for each class at a given age. The different trajectories are categorized into Trajectory 1: “always regular”, Trajectory 2: “early long transition”, Trajectory 3: “late long transition”, and Trajectory 4: “long to short transition.” The x-axis represents age (years) at menstrual cycle reports. The Bayesian Information Criterion (BIC) is displayed.

**Table 1:** Comparison of demographics and characteristics based on identified menstrual trajectory patterns in the Growing Up Today Study (GUTS) (1996–2023)

n	Overall	Trajectory 1 “always regular”	Trajectory 2 “early long transition”	Trajectory 3 “late long transition”	Trajectory 4 “long to short transition”	p-value^a^
	9,332	8,794	237	282	19	
Age at baseline (y), mean ± SD	12.2 ± 1.9	12.2 ±1.9	12.6 ± 1.9	11.9 ± 1.7	12.1 ±1.7	0.0003
Birth year, n (%)						
1980–1984	2,999 (32.1)	2.812 (32.0)	62 (26.2)	118 (41.8)	7 (36.8)	< .0001
1985–1989	4,070 (43.6)	3,824 (43.5)	108 (45.6)	130 (46.1)	8 (42.1)	
1990–1995	2,263 (24.3)	2,158 (24.5)	67 (28.3)	34 (12.1)	4 (21.1)	
Cohort, GUTS 1, n (%)	5,912 (63.4)	5,541 (63.0)	123 (51.9)	233 (82.6)	15 (79.0)	< .0001
Race and ethnicity, n (%)						0.79
Non-Hispanic white	8,769 (94.8)	8,265 (94.8)	221 (94.9)	264 (93.6)	19 (100.0)	
Non-Hispanic black	58 (0.6)	56 (0.6)	1 (0.4)	1 (0.4)	0 (0)	
Hispanic	157 (1.7)	149 (1.7)	3 (1.3)	5 (1.8)	0 (0)	
Non-Hispanic Asian	95 (1.0)	87 (1.0)	5 (2.2)	3 (1.1)	0 (0)	
Other	170 (1.8)	158 (1.8)	3 (1.3)	9 (3.2)	0 (0)	
Height in adulthood (cm), mean ± SD	160.2 ± 6.8	160.2 ± 6.8	160.6 ± 6.9	160.6 ± 6.5	162.0 ± 5.9	0.33
Childhood underweight^b^, n (%)	848 (9.1)	795 (9.0)	19 (8.0)	32 (11.4)	2 (10.5)	0.54
Childhood obesity^c^, n (%)	781 (8.4)	720 (8.2)	21 (8.9)	38 (13.5)	2 (10.5)	0.02
Adulthood underweight^d^, n (%)	784 (8.4)	731 (8.3)	22 (9.3)	26 (9.2)	5 (26.3)	0.04
Adulthood obesity^e^, n (%)	2,432 (26.1)	2,248 (25.6)	76 (32.1)	103 (36.5)	5 (26.3)	< .0001
Low strenuous physical activity (< 2hr/wk), n (%)^f^	3,317 (43.3)	3,128 (43.5)	73 (36.1)	111 (43.5)	5 (26.3)	0.09
High strenuous physical activity (> 11hr/wk), n (%)^f^	306 (4.0)	277 (3.9)	12 (5.9)	15 (5.9)	2 (10.5)	0.08
Age at menarche, mean ± SD	12.8 ± 1.1	12.8 ±1.1	12.8 ±1.2	12.6 ±1.1	12.8 ±1.2	0.04
Ever oligo-amenorrhea^g^, n (%)	3,165 (33.9)	2,664 (30.3)	235 (99.2)	251 (89.0)	15 (79.0)	< .0001
Menstrual characteristics^h^, n (%)						< .0001
< 21 days	1,159 (2.4)	1,019 (2.2)	34 (2.9)	85 (5.1)	21 (18.9)	
21–39 days	41,583 (85.8)	39,905 (87.6)	502 (42.9)	1,119 (67.3)	57 (51.4)	
>=40 days	3,918 (8.1)	3,590 (7.9)	120 (10.3)	182 (10.9)	26 (23.4)	
No periods	1,834 (3.8)	1,036 (2.3)	513 (43.9)	278 (16.7)	7 (6.3)	
Parity as of 2019, nulliparous, n (%)^f^	4,201 (58.4)	3,918 (58.3)	148 (74.0)	124 (49.2)	11 9 (61.1)	< .0001
Age at first pregnancy^i^ (y), mean ± SD	28.9 ± 3.6	28.9 ± 3.5	27.5 ± 4.0	28.5 ± 3.5	27.5 ± 5.1	0.008
Oral contraceptive use, ever, n (%)	8,487 (91.0)	7,975 (90.7)	225 (94.9)	270 (95.7)	17 (89.5)	0.004

GUTS: Growing Up Today Study.

a P-value was calculated by using the Kruskal-Wallis nonlinearity test for continuous variables, and chi-square test for categorical variables.

b Childhood underweight is defined using the CDC BMI-for-age percentile, with underweight classified as less than the 5th percentile for individuals younger than 20 years old.

c Childhood obesity is defined as obesity based on the International Obesity Task Force criteria for individuals younger than 20 years old.

d Adulthood underweight is defined as having a body mass index (BMI) of less than 18.5 for individuals aged 20 years and older.

e Adulthood obesity is defined as having a body mass index (BMI) of 30 or greater for individuals aged 20 years and older.

f Percentages were based on respondents only, excluding non-respondents.

g Ever oligo-amenorrhea is defined as having, at least once, reported a menstrual cycle length of 40–50 days, 51 days or more, cycles too irregular to estimate, or no menstrual period between 2003 and 2023.

h Percentages were based on all menstrual cycle length reports among the eligible participants.

i The average age at first pregnancy was calculated among participants who were parous in 2019.

**Table 2:** Comparison of conditions and characteristics based on identified menstrual trajectory patterns in the Growing Up Today Study (GUTS) (1996–2023)

n	Overall	Trajectory 1 “always regular”	Trajectory 2 “early long transition”	Trajectory 3 “late long transition”	Trajectory 4 “long to short transition”	p-value^a^
	9,332	8,794	237	282	19	
**Ovulatory disorder-related characteristics**						
Possible functional hypothalamic amenorrhea^b^, n (%)	302 (3.2)	254 (2.9)	27 (11.4)	18 (6.4)	3 (15.8)	< .0001
PMOS associated traits^c^, n (%)	3,105 (33.3)	2,892 (32.9)	71 (30.0)	131 (46.5)	11 (57.9)	< .0001
**Endocrine/Metabolic Conditions**						
PMOS self-report, n (%)	869 (9.3)	787 (9.0)	29 (12.2)	52 (18.4)	1 (5.3)	< .0001
Estimated age at PMOS diagnosis, mean ± SD	23.9 ± 5.6	23.8 ± 5.6	22.4 ± 6.2	25.8 ± 5.4	26.6	0.05
Severe acne in adolescence^d^, n (%)^e^	1,064 (11.8)	987 (11.6)	23 (10.1)	49 (18.3)	5 (26.3)	0.001
Hirsutism^f^, n (%)^e^	1,851 (22.0)	1,740 (22.0)	43 (18.9)	60 (21.8)	8 (42.1)	0.12
Maternal self-reported PMOS, n (%)	803 (8.6)	762 (8.7)	13 (5.5)	28 (9.9)	0 (0)	0.15
Hypothyroidism self-report, n (%)	897 (9.6)	818 (9.3)	34 (14.4)	43 (15.3)	2 (10.5)	0.0006
Hyperthyroidism self-report, n (%)	75 (0.8)	68 (0.8)	2 (0.8)	4 (1.4)	1 (5.3)	0.10
**Gynecologic Conditions**						
Endometriosis self-report, n (%)	551 (5.9)	496 (5.6)	16 (6.8)	38 (13.5)	1 (5.3)	< .0001
Fibroids self-report, n (%)	170 (1.8)	155 (1.8)	5 (2.1)	10 (3.6)	0 (0)	0.15
Medical Comorbidities						
Hypertension self-report, n (%)	717 (7.7)	665 (7.6)	18 (7.6)	33 (11.7)	1 (5.3)	0.08
Diabetes Mellites self-report, n (%)	158 (1.7)	149 (1.7)	5 (2.1)	4 (1.4)	0 (0)	0.87
Hypercholesteremia self-report, n (%)	1,326 (14.2)	1,230 (14.0)	31 (13.1)	65 (23.1)	0 (0)	< .0001

GUTS: Growing Up Today Study; PMOS: Polyendocrine metabolic ovarian syndrome.

a P-value was calculated by using the Kruskal-Wallis nonlinearity test for continuous variables, and chi-square test for categorical variables.

b Possible functional hypothalamic amenorrhea is defined as ever oligo-amenorrhea report between 2003 and 2023, underweight in childhood or adulthood, strenuous physical activity longer than 11hr/week, and not exhibiting PMOS traits, hypothyroidism or hyperthyroidism.

c PMOS traits are defined as self-reported PMOS diagnosis, hirsutism, or adolescent severe acne.

d Severe acne is defined as self-reported severe acne or oral contraceptive use due to acne under the age of 19.

e Percentages were based on respondents only, excluding non-respondents.

f Hirsutism is defined as simplified Ferriman-Gallyway score 4 or greater (Hair at upper abdomen, lower abdomen and chin).^26^

## Data Availability

The data analyzed in this manuscript cannot be shared publicly due to individual participant privacy. Investigators wishing to access the data may contact the data custodians directly at nhsaccess@channing.harvard.edu.
